# Improving access to psychological treatments: Lessons from developing countries

**DOI:** 10.1016/j.brat.2011.06.012

**Published:** 2011-09

**Authors:** Vikram Patel, Neerja Chowdhary, Atif Rahman, Helen Verdeli

**Affiliations:** a Department of Nutrition and Public Health Intervention Research, London School of Hygiene and Tropical Medicine, UK; b Sangath, India; c LSHTM, UK; d Department of Mental Health and Well-Being, University of Liverpool, UK; e Department of Counseling and Clinical Psychology, Teachers College, Columbia University, New York, USA

**Keywords:** Psychological treatment, Developing country, Depression

## Abstract

Even though psychological treatments have been advocated as treatments for a range of mental disorders by the WHO for scaling up through primary care globally, the vast majority of potential beneficiaries are unable to access these treatments. Two major barriers impede the path between evidence based treatments and improved access: the lack of skilled human resources and the acceptability of treatments across cultures. This essay synthesizes the experiences of programs which developed and evaluated psychological treatments for depression in three resource poor developing countries. These programs addressed the human resource barrier by training lay or community health workers to deliver the treatments and addressed the acceptability barrier by systematically adapting the treatment to contextual factors. All programs demonstrated significant benefits in recovery rates when compared with usual care demonstrating the effectiveness of the approach. The implications for these experiences to improving access to psychological treatments in the global context are discussed.

## Highlights

► Three trials in developing countries addressed barriers to improving access to psychological treatments for depression. ► The barrier of human resources was addressed by using lay health workers. ► A systematic process of adaptation was followed to ensure contextual appropriateness. ► Other barriers such as low recognition, low adherence and stigma were also addressed. ► The resulting interventions showed significant benefits when compared with usual care.

## The global ‘treatment gap’ for psychological treatments

The central role played by evidence based psychological treatments (PTs) in the management of mental illnesses is firmly established; in the global context, this importance has been reflected in the inclusion of a number of PTs for mental disorders in the recent WHO guidelines for the treatment of eight mental, neurological and substance use disorders in primary care settings (World Health Organisation, 2010). A rapidly growing evidence testifies to the large burden and adverse impact of mental illness in the global context and the efficacy and cost-effectiveness of specific treatments and delivery systems in low resource settings (Patel et al., 2007; Patel & Thornicroft, 2009). However, the reality is that the vast majority of persons living in such settings, most of which are in low and middle income countries (LMIC), do not have access to these treatments. A central concern of the field of global mental health is addressing this massive ‘treatment gap’, a term which is analogous to the concept of ‘coverage’ in public health, and which routinely exceeds 75% in most parts of the world (Kohn, Saxena, Levav, & Saraceno, 2004).

The challenges to implementing evidence-based PTs are enormous: several barriers have been identified ranging from inadequate investments in mental health care to stigma associated with mental illness (Saraceno et al., 2007). In this article, we focus on two barriers which we consider of critical importance in improving access to evidence-based PTs in low resource settings, viz. the lack of skilled human resources and the cultural acceptability and appropriateness of PTs (all of which have been developed in high-income countries (HIC) in different cultural contexts). We have based our observations on the experiences of three randomized controlled trials from three LMIC in Africa and Asia which sought to improve access to the WHO approved PTs for depression, viz., cognitive behaviour therapy-based (CBT-based) or interpersonal psychotherapy (IPT) (World Health Organisation, 2010). The authors of this article are lead investigators of these three trials.

## The trials

The characteristics of the three trials have been described in Table 1. The trials were carried out in rural community settings in Pakistan (the Thinking Health project or THP) and Uganda (the Group Interpersonal Psychotherapy for Uganda or IPT-GU) and in a mixed rural-urban primary care facility setting in India (the MANAS project). Although no precise estimates exist for lack of access to PTs in these settings, our experiences in the settings we describe in this paper would suggest that this may even exceed 90% of persons with depression. All three trials have published their primary results (Bolton et al., 2003; Patel et al., 2010b; Rahman, Malik, Sikander, Roberts, & Creed, 2008) as well as the methods to adapt the PT for the local context (Chatterjee et al., 2008; Rahman, 2007; Verdeli et al., 2004). The three trials are relatively large, with one being the largest ever trial for any mental disorder in a LMIC (Patel et al., 2010b) while another is the largest trial for maternal depression in LMIC (Rahman et al., 2008). Two trials evaluated the PT in a community setting, one of which was in group format (IPT) and one in individual format (CBT-based). The facility based trial had two PTs, one which was non-specific (psychoeducation) and one which was structured (IPT) and these were delivered in a collaborative stepped care format which included antidepressants. All three trials showed impressive benefits on improving recovery rates for depression, as well as on a variety of secondary outcomes (not shown in the Table, but reported in the primary publications).

We now turn to the experiences of the three projects in addressing the two major barriers to improving access to PTs.

## Addressing the shortage of human resources

The grave shortage of mental health professionals in LMIC and the inequities between and within nations in the availability and distribution of these resources has been well-documented(Saxena, Thornicroft, Knapp, & Whiteford, 2007). Put bluntly, relying on mental health professionals to deliver PTs will only address a tiny fraction of the treatment gap and task-shifting these interventions to more available and affordable members of the health workforce or community is widely acknowledged to be the only sustainable way of addressing this barrier (Patel, 2009; Patel et al., 2010a). Table 2 describes the types of human resources who delivered the PT in each of the three trials, their training and the supervisory arrangements. Several observations can be made from these findings. First, young women were the preferred therapists for two trials; in the third, therapist gender was matched for gender-specific groups, and most therapists were young adults. Second, none of the therapists had any prior training in mental health; in two trials, the therapist was a lay person from the community who had no health background at all. Third, the period of didactics varied but was, in general, relatively short, and focused on skills rather than theory and mostly involved participatory methods; the longest period (for the Goa trial) was due to the need to train the therapists in the overall collaborative care model and the actual PT focused session itself was for three weeks. Fourth, all the projects included a well-defined, structured supervisory protocol by a skilled mental health professional who played a variety of supervisory roles from case record reviews and, case discussions to joint case consultations. In summary, all three projects showed that people with no mental health background (or even no health background at all) and with relatively short training and continuing supervision could deliver the PT effectively.

## Addressing the acceptability and cultural appropriateness of ‘western’ psychological treatments

The cross-cultural applicability of PTs, all of which have been principally developed and evaluated in ‘western’ cultural contexts, to LMIC has been questioned by some authors (Tseng, April 2004). It is widely accepted that, just as with the classification and measurement of mental illnesses, mental health interventions also need cross-cultural adaptation to ensure the acceptability and appropriateness to the particular context (Patel, 2000). All three projects adopted systematic methodologies to address this barrier, approximating the steps of the Medical Research Council (UK) guidelines for the development of ‘complex’ interventions (Craig et al., 2008). In MANAS, the adaptation of the PT was part of the overall adaptation of all the components of the collaborative stepped care intervention and involved three distinct phases over 14 months: consultation with stakeholders involving 14 meetings with local, national and international stakeholders from the public, private and academic sectors to explore the feasibility of the proposed intervention; formative research with people affected by depression and health care providers in four public and four private primary health care facilities with quantitative process indicator data and qualitative data through in-depth interviews with doctors, facility staff, health counsellors and patients collected to explore feasibility and acceptability of specific components of the intervention; and piloting of the intervention in four primary health care centres involving interviews with 77 patients to explore their experiences of the intervention and reasons for non-adherence(Chatterjee et al., 2008). In the THP, the intervention was developed over a period of 12 months through: consultations with key stakeholders including in-depth interviews with 30 depressed mothers, four focus groups with purposively sampled 24 Lady Health Workers, and key-informant interviews with six primary care staff; and examination of data from an epidemiological study in which the investigators had explored psychosocial risk factors for perinatal depression in the same community (Rahman, 2007).In the IPT-GU, an initial draft of the adapted protocol was developed over four weeks based on data from an ethnographic study of depression in the region, input from researchers and, clinicians identified as site supervisors for the trial, and dialogue with local community leaders. This working document was modified substantially during the 10-day group facilitator training, based on the trainees’ feedback about whether and how each strategy and technique needed to be modified to fit the local culture (Verdeli et al., 2004). Further piloting was carried out to assess therapist competence. Thus, in all three cases, the development of the intervention involved multiple steps and mixed methods with a heavy reliance on qualitative methods. Data synthesis involved triangulation of these mixed methods derived data and theories to obtain an in-depth understanding of the issues likely to affect the feasibility, acceptability, effectiveness and sustainability of the intervention.

This systematic process demonstrated that most of the broad procedures and techniques of both IPT and CBT were cross-culturally applicable (Table 3). However, there were some very important modifications which were needed to improve the acceptability of the PT and enhance the feasibility of its use by non-specialist therapists. These included: ‘simplifying’ the language and content by removing the jargon associated with them; using pictorial materials for guiding therapists and patients; avoiding the use of psychiatric labels while recognizing that the symptoms were a reflection of the health consequences of stress or ‘life problems’; using contextually appropriate methods and metaphors to communicate the techniques and procedures associated with the PT such as using religious idioms; de-emphasizing some components of the PT which were found to be unacceptable or difficult to communicate, for example the domain of ‘interpersonal deficits;; and involving other individuals in the affected person’s social world, in particular, family members.

## Addressing other barriers to scaling up

A major barrier which needs to also be addressed, alongside increasing the availability and affordability of culturally appropriate PTs, is to enhance the demand for such interventions. Community awareness about depression and its responsiveness to PT, usually culturally appropriate language (as was done in IPT-GU), and pro-active case finding using contextually validated screening tools (as was done in MANAS) offer two specific strategies to address this barrier. Several other barriers were encountered during the development of the interventions (Table 4). Notable barriers were the low acceptability of PTs in contexts where local communities were unfamiliar with the use of ‘talking’ treatments for health problems; the stigma associated with accessing health care for ‘mental’ health problems; the competing work pressures and low motivation for health care workers; and the low adherence often due to the opportunity costs due to time taken to attend sessions and the direct costs of transport to health facilities. Table 4 summarises the key strategies taken by the project teams to address these barriers.

## Implications for improving access to PT at scale

The MANAS, THP and IPT-GU project experiences demonstrated unequivocally that persons with no mental health experience can effectively deliver PTs either in a stand-alone format or as part of a delivery system which includes other treatments, if five key conditions are met: first, that they are recruited from the local community; second, that they are provided with well-designed participatory training based on active learning principles; third, that the PT is suitably modified to address the skill set of such workers and to address contextual issues and barriers; fourth, that supervision is provided by persons with more extensive mental health experience and skills based on structured protocols for supervision, risk management and other aspects of clinical governance; and fifth, that the stigma associated with mental illness is addressed by using contextually appropriate concepts and integrating mental health with routine health care or existing community delivery systems (e.g. microfinance groups).

We consider five implications of these findings to improving access to PTs in the global context. *First*, provided the conditions outlined earlier are met, the potential human resource for delivery of PTs can now be expanded to include virtually any member of the community. In particular, future work could examine the role of volunteers and peers (for example, other persons who have recovered from depression, or mothers for post-natally depressed women) and traditional healers in those settings where they are widely accessed. *Second*, technological and practical flexibility is essential for the delivery of PTs. Thus strategies such as conducting sessions on the telephone, broadening the settings for PT delivery to include homes and community settings, and tapping into family and community resources enhance opportunities for non-medical interventions to facilitate recovery. *Third*, the adaptation of PTs can greatly benefit from a systematic methodology to identify and address barriers to acceptability and feasibility. Such a methodology requires participation of representatives of diverse stakeholder groups; adequately designed formative and pilot studies typically utilizing mixed methods; and a systematic review of the literature or qualitative studies on cultural expressions of distress and incorporating these findings in the proposed intervention. We are currently engaged in a new program (PREMIUM http://www.sangath.com/details.php%3fnav_id=123) which seeks to systematically ‘codify’ this methodology. *Fourth*, such a procedure shows that while some components of PT show universal applicability, others do need considerable adaptation for transferability to this context. Future work needs to define the extent to which such adaptations may influence the original theoretical model which lay at the heart of the PT and the extent to which elements common to many brief structured PTs (for e.g. goal setting, problem solving, improving interpersonal communication, etc) may out-weigh the differences between PTs. It is plausible that these common elements may be the most cost-effective interventions to improve access to PT in low resource contexts. *Fifth*, we think that much can be learned through sharing of knowledge about improving access in LMIC and HIC. There is a growing evidence base in HIC on improving access to PTs for ethnic minority groups who have traditionally been under-represented in PT services (Miranda et al., 2005). Many of the lessons drawn from those studies mirror the experiences we have described in LMIC and point towards some ‘universal’ truths about adapting PTs across cultures and health systems.

Ultimately, scaling up evidence-based PTs in low resource settings will also need to recognize that partnerships with, and commitments from, key stakeholder communities concerned with health care delivery is essential from the outset. Health systems in most LMIC are struggling to cope with great challenges ranging from the rising burden of chronic diseases to scarce resources; in this context, we believe that the inherent nature of PTs, unlike many more ‘medical’ interventions, afford the unique opportunity of a ‘bottom–top’ approach whereby the community could be much more actively involved and ‘empowered’. For this to happen, local and national health care agencies should be involved from the early stages of treatment adaptation and development to engage them in subsequent treatment uptake, not least by recognizing the value of PTs and ensuring appropriate resources for health workers engaged with delivery of PTs. Arguably, however, the biggest barrier to scaling up may be the perception held by mental health specialists about the risks of non-specialist health workers delivering PTs; the trials we have described have shown that using such providers led to acceptable, feasible and effective interventions for depression. Thus, a commitment by the global community of mental health professionals about the safety, effectiveness and necessity for ‘task-shifting’ of PTs to lay and community health providers is a pressing need. Above all, we need to emphasize that the central component of improving access is not simply handing over of the responsibility of delivering PT to less trained persons, but sharing these with a wider group of available human resources so as to ultimately close the ‘psychological treatment gap’ globally.

## Figures and Tables

**Table 1:** Characteristics of trials of psychological treatments in low and middle income countries (LMIC).

Trial	Country	Design	Target population and sample size	Psychological treatment and other active intervention	Comparison group	Primary outcome effects
MANAS	India	Cluster randomised controlled trial in 12 PHCs and 12 GP facilities	Adults over 17 years attending primary care facilities and screening positive for CMD	Psychoeducation, IPT, antidepressants and specialist referral delivered in a collaborative stepped care framework	Enhanced usual care – screening & antidepressant treatment guidelines for doctors	24% reduction in prevalence of depressive or anxiety disorder in participants with depression over 12 months in intervention arm in public facilities: RR = 0.76, (95% CI 0.59, 0.98; *p* = 0.04). No effect in private facilities
			2796 patients enrolled			
Thinking Healthy Programme (THP)	Pakistan	Cluster randomized controlled trial of women living in 40 Union Councils in two sub-districts	Married women aged 16–45 years, in the third trimester of pregnancy, meeting SCID criteria for DSM-IV major depressive episode	Psychological treatment incorporating cognitive and behavioural techniques delivered at home over 16 sessions starting from the last month of pregnancy until 10 months post-partum	Enhanced routine care, including a similar number of sessions	78% reduction in prevalence of depression at 6 months in intervention arm (AOR 0.22, 95% CI 0.14–0.36, *p* < 0.0001); 77% reduction at 12 months (AOR 0.23, 95% CI 0.15–0.36, *p* < 0.0001)
			903 mothers enrolled			
Uganda Interpersonal therapy (IPT-GU)	Uganda	Cluster randomized controlled trial in 30 villages	Adults ≥ 18 who: 1) were identified by others or self-identified with local syndromes equating to DSM-IV depression, and 2) screened positive for DSM-IV major depression or DD-NOS	IPT adapted for local population; delivered in 2 individual and 16 weekly group sessions	Information about using other locally available resources (e.g. local healers, NGO services)	In the intervention arm: 79.5% reduction in prevalence of depression at termination (4 months). Using adjusted difference in mean depression score change: AOR 13.91, 95% CI 10.99–16.84, *p* < 0.0001; 74.3% reduction in prevalence of depression at 6 months following termination; AOR 13.98, 95% CI 12.17–15.79, *p* < 0.0001
			248 participants enrolled			

**Table 2:** Characteristics of the ‘delivery agent’ for psychological treatments.

	MANAS	Thinking Healthy Programme	IPT-GU
Gender	Women	Women	Males and females matched by sex to groups (which were single-sex)
Age	Mean age: 27 years	Mean age: 25 years	Age range: most between 18 and 22 years
Educational qualifications	Mostly college graduates in any field with no health background	Mostly high school completers	Mostly high school completers, some enrolled in college
Role in the health system	Specifically recruited for the trial from the local community	Lady Health Workers part of the primary health care system	Hired by the NGO World Vision specifically for the trial
Duration of training	8 weeks for entire training of the MANAS stepped care intervention. 3 weeks for PT training	2-day training workshop and 1-day refresher after 4 months; regular refresher sessions were included in monthly supervision, with emphasis on incremental experiential learning	10-day intensive, residential, “IPT boot camp” with group facilitators
Type of training	Participatory methodology; specific methods used were: manual; didactic lectures; small and large group discussions; discussion of scripts or case material; role-plays; patient materials	Participatory methodology involving: a training video; role-plays; discussions; manual; patient workbooks and materials. Experiential learning	Both didactic and experiential group process training based on IPT principles, strategies, and techniques, including lectures, modelling and role-plays
Supervisor characteristics	Mental health specialists with clinical experience in PT and certified as IPT trainers	Mental health specialists with clinical experience in PT	Psychologists experienced in group therapy
Supervision format	Individual supervision, initially once in two weeks, reduced to once a month, on-site in the clinics. In addition, transcripts of every IPT session reviewed by the supervisor. Group supervision once a month	Monthly supervision in groups of ten for half a day. Emphasis on experiential learning through shared experiences of the group	Weekly group and individual supervision. On-site supervisors had weekly phone supervision with IPT trainers based in New York

**Table 3:** Modifications to psychological treatments for use in these trials.

MANAS	Thinking Healthy Programme	IPT-GU
**Aspects of PT which did not need significant adaptation**		
The phases of IPT treatment; the interpersonal problem areas; techniques such as linking mood to interpersonal event, role-play, communication analysis and decision-analysis.	Therapeutic empathic relationship, collaboration with the family, homework, and monitoring of mood.	The phases of IPT treatment; the focus on emotion recognition and exploration, antidepressant strategies such as giving hope, linking depression to the relevant interpersonal problem areas (grief, interpersonal disputes, role transitions), teaching specific strategies to manage the interpersonal problems better, including: generating options to counter helplessness of depression, helping patients identify advocates, clarifying interpersonal expectations and improving communication, and breaking social isolation. Techniques such as linking mood to interpersonal event, role-play, communication analysis, decision-analysis, and homework.

**Aspects of PT which did need significant adaptation**		
Language: The sessions were structured in greater detail with simplified scripts in the local language. Concepts: explaining depression as a stress related illness rather than using the term ‘depression’ or any psychiatric label. Use of metaphors: mood ratings were elicited from patients by showing a picture of a mood ladder with each rung depicting a higher or lower level of mood intensity. Use of handouts: with simplified explanation of the psychological treatment for patients and family members. Exploration of the use of religious practices as a coping method. Delivery in the individual format since group IPT was not feasible mainly due to patients’ concerns regarding confidentiality and inability to return for regular weekly sessions.	Focus on mother and infant health rather than maternal depression to enhance acceptability. Use of terms ‘stressed’ or ‘burdened’ where necessary and avoidance of psychiatric labels. Involvement of significant family members in suggesting alternative healthy thinking. Designation of a “health corner” in each house, and a “health calendar” provided to each mother to monitor homework and chart progress. Having an a-priori agenda for intervention, set within the context of the perinatal period: – the mother’s personal health, the mother-infant relationship, and the psychosocial support of significant others. Using culturally appropriate illustrations, for example characters depicting mothers, infants and other family members, to aid guided discovery. Ensuring culturally appropriate homework activities, for example not expecting outdoor activities during the *chilla* (40-day confinement of mothers after delivery) when mothers do not go out of the house.	More structured therapy training to take into account the group facilitators’ lack of previous therapy experience (e.g. through the use of scripts). IPT language simplified, for example, grief renamed “death of loved one(s),” interpersonal disputes became “disagreements,” and role transitions became “life changes”. Use of single-sex groups to encourage disclosure. Use of local idioms of distress to discuss depression presentation and clarified that this was not “madness”. The interpersonal deficits problem area was removed based on feedback that people are involved daily in communal activities and social isolation is rare. Specific modifications within each of the other three problem areas to improve compatibility with the local culture, for example, in the grief problem area, given the multiple losses associate with HIV the emphasis was not on in-depth reconstruction of each relationship but on adjusting to life without the lost loved ones and breaking social isolation.

**Table 4:** Barriers to improving access to psychological treatments.

Challenge faced	How this was addressed
Low acceptability due to doubts about its usefulness or confidentiality of the discussions with the therapist	● Modifying the way the choice of IPT v/s ADT was offered to the patient and revising the introduction to IPT to emphasize its benefits (MANAS) ● Incorporating elements of IPT into the generic psychoeducation module (MANAS) ● Reinforcing the value of IPT with primary care doctors who encouraged patients to opt for this treatment (MANAS) ● Selection of existing health workers as delivery agents who already have a relationship with the client group (THP) ● Training of health workers to engage with other family members (THP) ● Using infant health and development as the ‘agenda’ for engagement (THP) ● Using local metaphors to convey the idea that this special type of talking would help build skills rather than providing a temporary solution to address the expectation of material goods (IPT-GU) ● Holding groups in community settings to generate a sense of familiarity and ease (IPT-GU)
Low patient adherence	● Expanding the psychoeducation module to *3 sessions* in which key components of IPT were incorporated and offered to all patients with CMD at step 1 of the stepped care intervention (MANAS) ● Use of flexible appointments (MANAS) ● Use of telephone counselling (MANAS) ● Integrating the sessions with routine home-visits of the LHWs (THP) ● Linking the therapy with infant health which was perceived to be a tangible outcome by the family (THP)
Motivation of health workers	● Integrating the intervention into routine day to day work of the LHW (THP) ● Improving communication skills which helped general work (THP) ● Group support through monthly supervision meetings (THP) ● Positive feedback and ‘trouble-shooting’ through supervision (MANAS, THP, IPT-GU) ● Hiring the lay IPT-GU facilitators (by the NGO World Vision) at the end of trial (IPT-GU)
Interference with existing health system	● Highlighting positive influence of intervention on general infant health outcomes (THP) ● Improving communication skills of health workers (THP) ● Highlighting positive influence on primary health care patient outcomes through engagement with PHC doctors (MANAS) ● Provision of additional human resource (MANAS)
Stigma	● Using infant health and development as the ‘agenda’ for engagement (THP) ● Combining routine home-visits with PT sessions ● Using “stress” and “burdened”, or locally appropriate terms, as alternative expressions for ‘depression’ (MANAS, THP, IPT-GU) ● Explaining the purpose of the groups and the types of themes discussed (e.g. promoting harmony in the household) provided to the community (IPT-GU)
